# The myth of menstruation: how menstrual regulation and suppression impact contraceptive choice

**DOI:** 10.1186/s12905-019-0827-x

**Published:** 2019-10-28

**Authors:** Andrea L. DeMaria, Beth Sundstrom, Stephanie Meier, Abigail Wiseley

**Affiliations:** 1 Department of Public Health, College of Health and Human Sciences, Purdue University, 812 West State Street, West Lafayette, IN 47907 USA; 2 Department of Communication, College of Charleston, Charleston, SC USA; 3 Division of Consumer Science, Purdue University, West Lafayette, IN USA; 4 College of Health Professions, Medical University of South Carolina, Charleston, SC USA

**Keywords:** Menstruation, Contraceptive decision-making, LARC, Mixed-methods

## Abstract

**Background:**

Women in the US have access to various hormonal contraceptive methods that can regulate menstruation. This study examined the attitudes and perceptions of reproductive-aged women toward contraceptive methods, including how menstrual regulation and suppression preferences influenced contraceptive choice.

**Methods:**

Data collection used a mixed-methods approach, including 6 focus groups (*n* = 61), individual interviews (*n* = 18), and a web-based survey (*n* = 547).

**Results:**

Participants described contraceptive method preferences that allowed monthly bleeding and daily control, expressing concerns about long-acting reversible contraception (LARC) because of decreased user involvement. Some participants noted LARC improved their menstrual control. Many participants felt menstruation was healthy, whereas suppression was abnormal and resulted in negative health outcomes. Though participants indicated LARC as beneficial (M = 4.99 ± 1.66), convenient (M = 5.43 ± 1.68), and healthy (M = 4.62 ± 1.69), they chose combined oral contraceptives due to convenience.

**Conclusions:**

Findings suggest women need more information about menstrual regulation and suppression before selecting a contraceptive method, specifically in relation to LARC versus combined oral contraception. Framing menstrual suppression as healthy and natural may improve perceptions of long-term health consequences related to LARC. Providers should discuss menstrual suppression safety to ensure selection of contraceptive options aligning with women’s preferences and needs.

## Background

Menstruation may increase risks for some cancers, endometriosis, and anemia [1–3]. Prior literature suggests pre-industrial women menstruated approximately 100 times/lifetime due to extended pregnancy and breastfeeding periods; however, contemporary Western women menstruate nearly 400 times [2, 4]. Research demonstrated more frequent menstruation may counteract protective effects of high parity and late menarche [4–6]. Frequent menstruation may increase ovarian, endometrial, and breast cancer risk [7] and menstruation-related symptoms, such as heavy bleeding, dysmenorrhea, pre-menstrual syndrome, and endometriosis, among others [7–9]. Hormonal contraception, particularly combined oral contraceptives (COC), protect against osteoporosis, pelvic inflammatory disease, and certain gynecological cancers [7, 10, 11]. Women in the US have access to various hormonal contraceptive methods, including COCs and long-acting reversible contraceptives (LARC) (e.g., intrauterine devices (IUD) and implant), that can regulate menstruation [8, 12, 13]. The majority of US women rely on COC as their primary contraceptive method, which is up to 99% effective at preventing pregnancy with perfect use, but has a 9% typical use failure rate [12, 14]. LARC methods are more effective at preventing pregnancy, longer lasting, and do not depend on perfect adherence like COCs [12–14].

Contraceptive choice may also relate to factors including cost, knowledge, access, and side effects, like menstrual suppression [15, 16]. While all hormonal birth control methods can manipulate a woman’s menstrual cycle, their means vary [17]. Some COCs allow for monthly bleeding, while LARC methods can cease menstruation completely [18]. COCs are primarily discussed as an option for menstrual regulation and suppression; however, the hormonal IUD and implant may also reduce or cease monthly menstruation [7, 8, 18]. Thus, LARC methods are beneficial to women who desire menstrual regulation. Despite research on menstrual manipulation safety, myths and misperceptions remain regarding COCs and LARC [15]. The COC 21/7 regimen was designed for women and providers who believe monthly bleeding is natural and healthy [2, 5]; yet, this bleeding results from hormonal withdrawal, not from the biological cycle, and offers no medical benefits [7, 19].

Existing research presents conflicting information on women’s menstrual suppression preferences. Some researchers have documented women prefer few to no menses [20–22], particularly in American and European populations [23]. Lakehomer et al. [20] found that 65% of female college students preferred to menstruate less than monthly. Ferrero et al. [24] examined menstruation attitudes and found less frequent menstruation was important in enhancing daily life. Other documented reasons for menstrual suppression included convenience and relief from menstrual discomfort [1, 25, 26]. Snow et al. [27] illustrated women may prefer complete menstrual suppression for reasons including pain and stress relief, which greatly impact women’s health and life quality [28].

Alternatively, studies noted women hold negative menstrual suppression attitudes [29, 30], believing regular menses is natural and provides evidence of pregnancy prevention [1, 21, 25, 27, 31]. A recent study demonstrated nearly half of participants believed monthly menses were necessary to rid the body of menstrual build-up [1]. Szarewski and Moeller [1] found over 40% of participants believed reducing menstrual frequency resulted in negative outcomes, such as infertility or weight gain.

Conflicting literature emphasizes the need for further research on women’s attitudes toward menstrual suppression and contraceptive use [23]. Sundstrom et al. [32] described conflicting preferences among postpartum women regarding menstrual regulation via hormonal contraception, with some women desiring regulation and suppression to avoid menstrual pain and discomfort and others expressing concerns about negative health outcomes. Gunson [33] noted safety considerations associated with extended hormonal menstrual manipulation, but factors such as pain relief and increased contraceptive options also impacted women’s suppression preferences. Critiques of suppression related to social norms surrounding menses as an illness to be managed with medication and contraception [34]. However, women also identified inclusive narratives, in which menstruation perceptions could encompass natural menstruation and hormonal regulation and suppression [19, 35, 36]. Wisely et al. [37] noted in a conference paper presented at the American Public Health Association Annual Meeting the need to further examine women’s menstruation-related contraceptive needs, detailing this gap.

Considering the significance of preferences in menstrual management [36], understanding how menstrual needs impact contraceptive choice is vital to delivering high-quality healthcare. As such, this study examined reproductive-aged women’s menstrual regulation and suppression attitudes and how these influenced contraceptive choice.

## Methods

As part of a larger study on LARC knowledge and attitudes, the current study used a mixed methods approach consisting of three phases: 1) focus group discussions; 2) individual interviews; and 3) web-based survey. Women aged 18 to 44 living in or near an urban southeast coastal region of the US were recruited to participate through online advertisements that appeared in Facebook newsfeeds, electronic advertisements on a primary local newspaper website, individual emails, and printed flyers. Informed consent was obtained from focus group and interview participants, and implied consent was obtained from survey participants, prior to study participation. Methods and procedures for this study were approved by the primary author’s institutional review board.

### Qualitative measures: focus groups and interviews

Six focus groups were conducted between March and April 2014; each included 8 to 12 women, with a total of 61 participants. Discussions lasted approximately 2 hours. Participants received a $50 incentive, parking compensation, and refreshments for their time and efforts. All discussions were recorded using the SoundNote iPad application. All moderators and co-moderators received graduate-level qualitative research methodology training.

Focus group discussions followed a semi-structured guide consisting of a pre-determined list of questions that allowed the moderator to adapt and/or rearrange questions, and clarify topics to enhance conversation (Additional file 1). The questions investigated participants’ general contraceptive method knowledge and contraceptive decision-making attitudes. Participants were asked questions related to the theory of planned behavior (TPB) [38] construct *attitude*, such as “have you heard of: the implant; the IUD (intrauterine device)?” and “what do you think about these methods?” Additionally, focus group questions assessed *subjective norm*, (e.g., “whose opinion most influences your contraceptive choice?” and “do the people in your life support your contraceptive choices?”) and *perceived behavioral control* (e.g., “how much do you think you are in control of choosing your contraceptive method?”).

During April 2014, 18 individual interviews were conducted, lasting approximately 1 hour. Participants received a $25 incentive and parking compensation for their time and efforts. All interviews were recorded using the SoundNote iPad application. Researchers trained in graduate-level qualitative methodologies conducted interviews. Interviews followed a semi-structured protocol to encourage a conversational partnership (Additional file 2). The interviews investigated the participants’ knowledge of and experiences with various contraceptive methods, with questions related to attitude and subjective norm (e.g., “do people in your life support your contraceptive choices?” and “do you know anyone that uses LARC methods? What have they told you about their experiences with these methods?”). Additionally, questions explored women’s perceived behavioral control regarding LARC (e.g., “would you consider switching to a nondaily or LARC method? If so, when?”).

### Quantitative measures: web-based survey

A web-based survey was used to reach a larger sample. In total, 547 women completed the 15-min survey during June and July 2014. Participants were eligible to enter their name into a drawing to win one of three $100 incentives. The survey consisted of questions related to demographics, the TPB, contraceptive use, and reproductive health history. Demographic questions included age, race, ethnicity, education, and sexual orientation.

The TPB was used to assess attitudes, subjective norms, perceived behavioral control, and intention toward LARC uptake. Statements related to the TPB used a seven-point bipolar adjective scale. For example, “choosing the IUD or implant as my primary birth control method would be …” had answers ranging from: 1 = “extremely unhealthy” to 7 = “extremely healthy” in order to assess attitude. Additionally, items assessed subjective norm (e.g., “the people in my life whose opinions I value would support my decision to use an IUD or arm implant as my primary birth control method: extremely unlikely/extremely likely”) and perceived behavioral control (e.g., “I have sufficient information to decide if an IUD or implant is right for me: strongly disagree/strongly agree.”). Contraceptive use questions determined the participants’ familiarity with various contraceptives (e.g., “why did you choose this as your primary contraceptive method?”).

### Data analyses

Focus group and individual interviews were transcribed verbatim. Grounded theory methodology provided the data analysis framework and an inductive approach to data analysis privileging participants’ perceptions, stories, and experiences. Corbin and Strauss’s [39] extension of grounded theory allows for incorporation of existing theory; thus, researchers utilized the TPB as a framework to initiate data analysis and compare findings from the data to theoretical constructs. A constant comparative method was used throughout data collection and analysis to compare across and within focus group and interview transcripts to identify patterns and themes. Focus group and interview data were coded independently using HyperRESEARCH 4.0.1 qualitative data analysis software. Researchers conducted line-by-line open and axial coding to develop conceptual categories and used in-vivo codes developed from participant words to further understand menstrual attitudes and beliefs, incorporating these into emerging themes. Regular research meetings allowed for discussion of emergent themes. Descriptive statistics were utilized to analyze participant characteristics and survey response items. All quantitative analyses were performed using IBM-SPSS 21.0.

## Results

### Qualitative results: focus group and interview findings

Qualitative analysis revealed three themes related to perceptions of menstrual suppression when choosing a contraceptive method. Specifically, themes emerged regarding perceived increased menstrual control with COCs compared to LARC methods, the benefits of menstrual regulation and suppression through LARC methods, and the myth of menstruation demonstrated by the influence of withdrawal bleeding associated with COCs. Each theme is provided with illustrative quotes in this section. Participant demographics are located in Table 1.

**Table 1:** Qualitative and quantitative participant demographics

Variable	Phases 1 & 2	Phase 3
Race		
White, Non-Hispanic	67 (84.8)	376 (68.70)
Black or African American	12 (15.2)	43 (7.90)
Other	0 (0)	36 (6.60)
Born in US	–	432 (79.00)
Age	23.63 ± 5.40	24.41 ± 6.02
18 to 24 years	56 (70.89)	289 (52.80)
25 to 34 years	20 (25.32)	129 (23.60)
35 to 44 years	3 (3.80)	37 (6.80)
Sexual Orientation		
Heterosexual	72 (91.14)	405 (74.00)
Homosexual/Bisexual/Asexual/Intersex/Queer	7 (8.86)	49 (9.00)
Relationship Status		
Single	46 (58.23)	173 (31.60)
In a Relationship	33 (41.77)	281 (51.40)
Sexual Relationship Status		
Exclusive/monogamous sexual relationship	–	281 (51.40)
Sexually active, not monogamous	–	53 (9.70)
Not currently/never has been sexually active	–	120 (21.90)
Education		
High school diploma/GED	2 (2.53)	36 (6.58)
Some college education/undergraduate education	56 (70.89)	314 (57.40)
Some graduate education/graduate degree	21 (26.58)	104 (19.01)
Insurance Coverage		
Private	68 (86.1)	354 (64.70)
Government	4 (5.06)	59 (10.80)
None	7 (8.86)	41 (7.50)
Received Insurance Coverage Due to ACA	12 (15.2)	47 (8.60)
Income		
Less than $30,000	27 (34.18)	102 (18.60)
$30,000 to $69,999	12 (15.19)	139 (25.40)
$70,000 or more	24 (30.38)	171 (31.30)

Listed as *n* (%). Frequencies that do not sum to total represent missing data

#### Menstrual control with COCs versus LARC methods

Some participants preferred COCs to LARC because they believed COC provided more menstrual control. One participant perceived using a COC would provide her the greatest cycle regulation because “I have irregular periods, and a hormone would help regulate that.” This draws attention to misunderstandings about the hormonal benefits of LARC, which are similar to COC. Another common concern among participants was lack of control. One participant noted, “I know it’s on me to take [the COC] every day. And, let’s say I forget it for a day … I know that [I missed it] versus if [the IUD] might have slipped out.” Similarly, another participant stated while discussing the implant, “I just wouldn’t want something in me for that long. Like five years. I’d just rather take mine out monthly just so I know it’s taken out and then have a week off. I just wouldn’t want it in that long.” This participant felt being able to have a monthly menses through vaginal ring removal allowed more control and assurance than LARC provides. Participants felt more confident about non-LARC because they could control if and when menstruation occurred. Some participants felt COC provided more menstrual control and was a more comforting option because of user regulation.

#### The benefits of menstrual regulation and suppression through LARC methods

While some participants were hesitant about LARC benefits, other participants felt positively about menstrual suppression as a possible side effect. Participants who felt positively about LARC menstrual suppression saw the long-acting mechanism as beneficial for several reasons (e.g., convenience, efficacy, symptom relief). One participant, who currently had an implant, felt it had “been a great option because in college your schedules are crazy and you forget to take [the COC] and you don’t want to replace the ring every three weeks. And, it’s just an easy, kind of, no brainer.” She noted the benefits of long-term options, “… frankly as a society we have gotten lazy; we want instant gratification, and I think the closest thing to that in birth control is long term effects, rather than [the COC].”

Monthly menses suppression due to LARC methods was another benefit that enabled participants to have control over their bodies. Participants with LARC experience supported menstrual suppression associated with the IUD and implant. One participant “liked that I did not have a period at all for five years.” Another spoke about a friend who used LARC, saying, “[she] said it was worth it because she knew for five years this is 100% what was going to happen with her body. She knew exactly when her menses was gonna be. She knew she wasn’t gonna get pregnant. She was in control.” Menstrual suppression can also help with menstrual symptoms. One participant used an IUD to combat endometriosis, recognizing a reduction in “difficulties with cramping.” Another participant said people using the implant “say that it’s awesome. They don’t have a period anymore, and they have no acne and everything is great.”

#### The myth of menstruation: the influence of withdrawal bleeding associated with COCs

Several participants believed monthly menstruation, either through natural means or withdrawal bleeding, was healthier than menstrual suppression resulting from LARC. One participant worried, “if I’m not getting my period I just don’t find that to be natural, and that would make me worry … so I probably wouldn’t be comfortable with the IUD.” Another participant stated, “some of those [COCs] [that advertise] ‘You’ll only have four periods a year!’ … It’s just kind of odd. And then these doctors are like, ‘You don’t actually have to have your period every month!’ I just don’t really believe it … it just kind of freaks me out.” Additionally, one participant felt having a menses every 3 months “just does not seem healthy” and that the “body needs to get that out once every month.” Participants expressed apprehension about suppressing or limiting menses, and these concerns affected their choice of contraception.

Another participant, who was currently using COCs that did not follow the 21/7 regimen and did not induce monthly withdrawal bleeding, was disconcerted with lack of menses. She felt “like I’m over this. I want my period. So I stopped taking it and now I’m back to getting regular periods.” Because she was sexually active and used monthly menstruation as a reassurance she was not pregnant, menstrual suppression was concerning. A second participant argued that even COCs were unhealthy since it was “putting something into your body when your body has a natural cycle that it goes through.” This suggests lack of knowledge and awareness surrounding menstruation and withdrawal bleeding. Participants were also concerned about menstrual suppression with the implant. One participant wondered, “how can you tell if you’re pregnant?” Concerns about menstrual suppression risk appeared as barriers to choosing LARC methods over COCs.

### Quantitative results: web-based survey findings

#### Participant demographics

Demographic data for participants is located in Table 1.

#### The theory of planned behavior

Participants answered bi-polar scaled items ranging from one (negative association) to seven (positive association). Results indicated obtaining an IUD or implant would be slightly frightening (M = 3.74 ± 1.66) and slightly painful (M = 3.68 ± 1.50). However, participants perceived choosing an IUD or implant as beneficial (M = 4.99 ± 1.66), convenient (M = 5.43 ± 1.68), and healthy (M = 4.62 ± 1.69). Participants indicated a slight agreement (M = 4.70 ± 2.03) with the statement, “I have sufficient information to decide if an IUD or implant is right for me.” While participants appeared to be knowledgeable about LARC and have positive attitudes toward these methods, fear related to IUD or implant insertion or side effects may pose a barrier to LARC use.

#### Contraceptive use

The most common contraceptive methods participants had ever used were the COC (71.84%; *n* = 393) and condoms (73.31%; *n* = 401) (see Table 2). Approximately two-fifths of participants (40.40%; *n* = 221) used the withdrawal method. Less than one-third of participants (31.99%; *n* = 175) had ever used emergency contraception. Some participants had ever used an IUD (10.79%; *n* = 59), shot (9.51%; *n* = 52), or vaginal ring (8.96%; *n* = 49). Few participants indicated using the patch (4.20%; *n* = 23), the implant (2.37%; *n* = 13), or female sterilization (1.10%, *n* = 6). Additionally, 5.48% of participants (*n* = 30) never used a contraceptive method.

**Table 2:** Quantitative contraception use

Variable	Total sample
Current Contraceptive Method	
Birth Control Pill	207 (37.80)
Condom	61 (11.20)
Withdrawal Method	221 (40.40)
Emergency Contraception	175 (32.00)
Implant (Implanon® or Nexplanon®)	11 (2.00)
IUD (Mirena®, ParaGuard®, Skyla®) Shot (Depo-Provera or Lunelle)	48 (8.80)
Natural Family Planning Method or Rhythm Method	5 (0.09)
Patch (Ortho Evra)	2 (0.40)
Shot (Depo-Provera or Lunelle)	9 (1.60)
Vaginal Ring	49 (9.00)
Female Sterilization	6 (1.10)
Never used a contraceptive method	30 (5.50)
Reason for primary contraceptive method	
Convenience of use	123 (22.50)
Availability of method	46 (8.40)
Lack of information about alternatives	10 (1.80)
Unreliability of alternative methods	24 (4.40)
Reduce side effects (cramping, acne, etc.)	9 (1.60)

Listed as *n* (%)*.* Frequencies that do not sum to total represent missing data

Among the various contraceptive methods, the most common primary method was the COC (42.77%; *n* = 207). Less than one-tenth of participants (9.92%; *n* = 48) indicated the IUD as their primary method of contraception. Additionally, 2.27% of participants (*n* = 11) used the implant as their primary contraceptive. When asked why they chose their primary contraceptive method, almost one-third of participants (30.90%; *n* = 123) stated it was due to use convenience. The next most common reason was the availability of the method (11.56%; *n* = 46). Unreliability of alternative methods was a reason cited by 6.03% (*n* = 24) of participants, while 10 participants (2.51%) indicated their choice was due to a lack of information about alternative methods. Reduced side effects, including cramping and acne, was the reason for choosing a contraceptive method for 2.26% (*n* = 9) of participants.

LARC methods were not widely used among participants, and rates were below the national average [14]. Despite beliefs that LARC methods were convenient, almost one-third of participants chose their primary contraceptive method for convenience, which was greater than the number of participants who indicated using an IUD or implant. This suggests while participants may find LARC methods beneficial and convenient, other methods, such as the COC, are more conveniently available.

## Discussion

Aligning with previous research [31, 40], women viewed the COC as the norm, particularly its relationship with menstruation. Participants in this study equated COC use with greater control over menstruation, negotiating a complex understanding of responsibility and menstrual regulation. Taking the COC at the same time daily (or not) allowed women to feel in control of their menstrual cycle. Hormonal regulation via COC allowed women to choose when menstruation would occur while still assuring a negative pregnancy status. Though most women in this study did not report choosing a contraceptive method for non-contraceptive benefits, decisions regarding COC adoption related to the ability to regulate menstruation and maintain monthly menses, as noted in past studies (e.g., [36]). Versions of the COC, the hormonal IUD, and the implant each release progestin that can regulate or suppress menstruation; yet, participants remained wary of LARC benefits. This finding elaborates upon previous studies [10] indicating most women would choose to control or suppress menstruation through the COC but may not be aware of the same benefits offered by LARC.

Though survey results indicated women felt LARC seemed frightening and painful, focus group and interview participants noted the benefit of autonomy provided by LARC. In addition to convenience of not taking the COC every day, women noted positive valuations of menstrual regulation and control through LARC. Despite findings illustrating COC users were more aware of menstrual suppression [41], LARC users appreciated knowing when and if menstruation would occur. Furthermore, qualitative data demonstrated women adopting LARC found menstrual suppression more acceptable than women choosing a COC, possibly due to increased conversations about LARC mechanisms and effects with healthcare providers [8, 13]. LARC also provided added non-contraceptive advantages related to menstrual suppression. Suppressing menstruation reduced cramping and helped improve painful endometriosis symptoms. This finding extends previous research [10, 36, 42] indicating women suppressing menstruation through COCs often chose to do so because of menstrual-related symptom reduction. Prior research indicated women, especially women at risk for endometriosis or who desire to control painful menstrual symptoms, can safely use hormonal contraception for extended periods for menstrual regulation and suppression, suggesting satisfaction and acceptability [6, 11, 42, 43]. Thus, addressing the beneficial effects of LARC on menstrual regulation may improve acceptability of both LARC methods and menstrual suppression.

Some participants viewed menstrual suppression favorably; however, most found the idea of not menstruating strange, unhealthy, and worrisome. In particular, women believed monthly bleeding demonstrated their body was functioning normally. This finding reflects previous research [33] that women worry about long-term health consequences of suppression related to infertility. Further, participants understood menstruation as indicators of fertility and reassurance of negative pregnancy status. The women in this study viewed COCs as healthy because they allow for a natural menstrual cycle, yet the ‘menstruation’ experienced on COCs is actually withdrawal bleeding, not menses [7, 8]. Thus, women remain unaware that bleeding experienced when taking a placebo is breakthrough bleeding and not an indicator of pregnancy or fertility status.

Despite increasing awareness from healthcare providers that menstruation is not necessary—only 7% of physicians feel menstruation is medically necessary—and many women’s desires for options to limit or prevent menstruation [44], women still choose contraception that maintain monthly menses. They express distrust and fear regarding methods allowing suppression, elaborating on prior literature [21, 35, 45]. Perceptions of monthly menstruation as healthy and necessary may reduce women’s autonomy to choose options that could improve their daily lives by reducing associated bleeding and frequently debilitating symptoms [2, 8, 35]; yet, it remains a social norm impacting women’s contraceptive decision-making even when women express a desire for dysmenorrhea and heavy bleeding relief [46]. Women continue to choose less effective contraceptive options due to concerns about menstrual suppression and perceptions that COCs are more natural and healthy. These perceptions may reduce women’s options should they desire to regulate or suppress menstruation, decreasing the opportunity to achieve their individual lifestyle needs and goals. Thus, changing women’s menstrual suppression perceptions, particularly among women who desire reduced menstrual frequency and management, necessitates addressing social norm barriers regarding monthly menstruation. During clinical consultations, providers should address the benefits of menstrual regulation and suppression, highlighting these as safe options for women, to reduce misinformation and knowledge disparities. Additionally, providers globally should discuss the monthly bleeding patterns associated with different contraceptive options to reduce health concerns and contraceptive discontinuation [23, 47]. This may improve the acceptability of contraceptive options, including LARC, that function to reduce menstrual frequency and may open up further options for women.

This study was not without limitations. First, as part of a larger study, participants were not directly asked about the effect of menstruation on contraceptive choice or explicitly questioned about attitudes toward menstrual suppression. Another limitation is the generalizability of this study to other populations, as participants resided in one region of the southeast. Additionally, data were limited to small cohorts of interview and survey participants in the US that may not reflect the experiences and opinions of geographically or demographically dissimilar populations.

Future research is needed regarding women’s menstruation knowledge, and the health benefits and risks of monthly menses, across global populations. Exploring social norm effects on preference of menstrual suppression should be examined. Further, future research should investigate whether women and healthcare providers discuss menstrual suppression in consultations and how women perceive these discussions. An evaluation of clinical care guidelines related to the topic could provide valuable insight into standard patient care. Additionally, menstruation importance in contraceptive choice should be further examined.

## Conclusions

This study provided insight into US women’s perceptions of menstrual suppression related to LARC. Menstruation-related factors may influence women’s contraceptive choices. Understanding menstruation preferences and experiences can ensure women’s individual concerns, beliefs, and needs are met, empowering women to make lifestyle-concordant contraceptive choices. Providers should explain suppression benefits to women who desire regulation in contraceptive counseling. Framing regulation and suppression as a healthy and natural choice may improve women’s acceptability. Providers should also be aware of common misperceptions related to LARC, including suppression, to effectively discuss contraceptive choices and potential barriers. Findings suggest women need more information about menstrual regulation and suppression before selecting contraception, specifically LARC. Discussing health benefits of menstrual regulation may address women’s fears, improving perceptions of long-term health consequences.

## Supplementary information

**Additional file 1.** Semi-Structured Focus Group Guide, Focus Group Questions and Probes to Investigate Contraception and Menstruation.

**Additional file 2.** Semi-Structured Interview Guide, Interview Questions and Probes to Investigate Contraception and Menstruation.

## Data Availability

The datasets used and analyzed during the current study are available from the corresponding author on reasonable request.

## Abbreviations

COC: Combined oral contraceptive pill
FDA: Food and Drug Administration
IUD: Intrauterine device
LARC: Long-acting reversible contraceptive
TPB: Theory of Planned Behavior
