# Supplementary material for: The myth of menstruation: how menstrual regulation and suppression impact contraceptive choice
Source: BMC Womens Health. 2019 Oct 28;19:125. doi: 10.1186/s12905-019-0827-x (PMC6816209; doi:10.1186/s12905-019-0827-x)
Supplement: Supplementary file 1 — Additional file 1. Semi-Structured Focus Group Guide, Focus Group Questions and Probes to Investigate Contraception and Menstruation. [file 12905_2019_827_MOESM1_ESM.docx]

**Focus Group Guide**

Okay! I would like to begin by finding out what your perceptions are on some important issues, such as health, and then I am going to show you message designs and concepts that were produced about contraception, to get your opinions about them.

So, the first couple of questions deal with health, and what health means to you.

1. Please use the paper and pen you were provided, and take a moment to write down what

comes to your mind when you hear the word *contraception or birth control.*

*(pause at least 20 sec)*

What were some things you wrote down?

*Probe:* How do you feel about contraception?

2. What are the different kinds of contraceptive methods you can think of that you know are out there for women to get?

*Probe: (If not mentioned)* Have you heard of: a. Condoms b. NuvaRing  c. Patch d. Shot e. Implant f. IUD (intrauterine device) g. Plan B (emergency contraception) h. Sterilization

*Probe:* What do you think about these methods?

*Probe:* Do you know anyone that uses these methods? What have they told you about their experiences with these methods?

*Probe:* How would you describe (*method*) to a friend who didn’t know much about it?

1. Please describe for me a time when you discussed contraception with people in your life? *Probe:* Do you discuss contraception with...Partner? Friends? Family? Mother/Aunts? Doctor?

*Probe:* Do the people in your life support your contraceptive choices?

*Probe:* Who is your most trusted source of information about contraceptive use? Why?

*Probe:* Whose opinion most influences your contraceptive choice? Why?

1. How much do you think you are in control of choosing your contraceptive method?

*Probe:* Has your health insurance paid for all of your birth control? Are there any methods that you thought about using but health insurance did not pay for?

*Probe:* Do you trust your health care provider’s advice on which method of contraception is best for you?

*Probe:* Do you feel that you chose your method based on his/her advice?

*Probe:* How do you feel about needing a prescription to obtain contraception?

1. Where do you go for more information about contraception? In other words, what sources of information do you use when you want to learn more about it?

*Probe:* Do you ask your doctor? Do you use media, like Internet or television or news?

*Probe:* How often do you use these sources?

*Probe:* Can you offer some examples of any contraception information you recently found?

*Probe:* Who or what is your most trusted source of information?

1. What messages have you seen or heard about contraception?

*Probe:* What did you think about those messages?

1. How would you most like to receive health messages regarding contraception?

*If participants don’t mention the following channels,*

*Probe:* Could you tell me about how, if at all, you use (or would like to use) ______________ to find out more information regarding contraception?

- Physician Counseling: Interpersonal Communication (OB/GYN, NP, etc.)
- Traditional written materials: books, magazines, brochures, etc.
- Videos/YouTube
  - *Probe:* How often do you visit YouTube? What type of videos do you watch?
- Skype
  - *Probe:* What do you think about Skype?
- Social networking sites (i.e. Facebook)
  - *Probe:* How often do you use Facebook? Who are you friends with on Facebook?  Who do you communicate with most often? What about a Facebook page grabs  your attention?
- Microblogging sites (i.e. Twitter)
  - *Probe:* How often do you use Twitter? Who do you follow on Twitter?
- Blogs
  - *Probe:* What type of blogs do you visit? How do you find these blogs? How frequently do you read these blogs?
- Mobile applications (i.e. apps on your cell phone)
  - *Probe:* What type of apps do you use right now? What apps do you use most frequently? What makes an app useful to you?
- Texts
  - *Probe:* How often do you text? Who do you text with (do you receive text messages from your doctor?)? What type of health messages would be most helpful to you regarding contraception? Do you have free text messages?

----------------------------------------

I want to show you some message concepts and designs, and then ask you to share your thoughts about the visuals, the statements, and any feelings or thoughts you have.

To help start our discussion, I would like you to use the paper and pen you have, and write down the first things that come to your mind from seeing these message concepts and designs.

So, the first concept... Now, write down anything you are thinking...(*pause 20 sec)*

1. Okay, what are some things you wrote down?
2. What do you think is the core message or one thing that you take away from the concept or design?

*Probe:* How well do you think the design met this goal with you?

1. What are your impressions about the characters or people used in the design?

*Probe:* Why do you think these particular characters were selected?

1. What audience, do you think, was the target for this message?

*Probe:* How well do you think you fit within the target audience?

1. What did you think about the slogan used in the design?

1. How did this concept make you feel about contraception? (nondaily and long-acting reversible contraception?)
2. In terms of emotions, how did this concept or message make you feel? In other words, how angry, frustrated, happy, pleased, or sad did you feel?

1. What, if anything, would you change about this message to increase use of nondaily or long- acting reversible contraception?
2. How, if at all, did viewing this message impact your opinion of these contraceptive methods?

*Probe:* Based on this message, would you be more likely to consider using a nondaily or LARC method?

*Probe:* Based on this message, do you plan to contact your health care provider to inquire about these options?

[Repeat 8-16 for each message concept and design]

17. How, if at all, would the sponsoring organization impact your assessment of the message concepts and designs you just saw?

*Probe:* Can you think of a sponsoring organization that would increase/decrease credibility of the message?

---------------------------------

18. So those are all the messages I have to share with you. Now that you have seen all four, how would you compare or contrast them in light of what they are trying to do?

*Probe:* How do they connect with you?

*Probe:* What is on your mind about contraception? (Would you consider switching to a nondaily or LARC method? If so, when?)

19. Those are all the questions I have. Can you think of other questions I should have asked, about the messages or your opinions on them, that I did not?

20. Are there any final comments you would like to share?
