# Supplementary material for: The myth of menstruation: how menstrual regulation and suppression impact contraceptive choice
Source: BMC Womens Health. 2019 Oct 28;19:125. doi: 10.1186/s12905-019-0827-x (PMC6816209; doi:10.1186/s12905-019-0827-x)
Supplement: Supplementary file 2 — Additional file 2. Semi-Structured Interview Guide, Interview Questions and Probes to Investigate Contraception and Menstruation. [file 12905_2019_827_MOESM2_ESM.doc]

**Interview Guide**

Okay! I would like to begin by finding out what your perceptions are on some important issues, such as health, and then I am going to show you message designs and concepts that were produced about contraception, to get your opinions about them.

So, the first couple of questions deal with health, and what health means to you.

1. Please use the paper and pen you were provided, and take a moment to write down what comes to your mind when you hear the word *contraception or birth control.*

*(pause at least 20 sec)*

What were some things you wrote down?

*Probe:* How do you feel about contraception?

2. What are the different kinds of contraceptive methods you can think of that you know are out there for women to get?

*Probe:* *(If not mentioned)* Have you heard of:

a. Condoms b. NuvaRing c. Patch

d. Shot e. Implant f. IUD (intrauterine device)

g. Plan B (emergency contraception) h. Sterilization

*Probe:* What do you think about these methods?

*Probe:* Do you know anyone that uses these methods? What have they told you about their experiences with these methods?

*Probe:* How would you describe (*method*) to a friend who didn’t know much about it?

3. Please describe for me a time when you discussed contraception with people in your life?

*Probe:* Do you discuss contraception with…Partner? Friends? Family? Mother/Aunts? Doctor?

*Probe:* Do the people in your life support your contraceptive choices?

*Probe:* Who is your most trusted source of information about contraceptive use? Why?

*Probe:* Whose opinion most influences your contraceptive choice? Why?

----------------------------------------

I want to show you some message concepts and designs, and then ask you to share your thoughts about the visuals, the statements, and any feelings or thoughts you have.

To help start our discussion, I would like you to use the paper and pen you have, and write down the first things that come to your mind from seeing these message concepts and designs.

So, the first concept…

Now, write down anything you are thinking…(*pause 20 sec)*

4. Okay, what are some things you wrote down?

5. What do you think is the core message or one thing that you take away from the concept or design?

*Probe:* How well do you think the design met this goal with you?

6. What are your impressions about the characters or people used in the design?

*Probe:* Why do you think these particular characters were selected?

7. What audience, do you think, was the target for this message?

*Probe:* How well do you think you fit within the target audience?

8. What did you think about the slogan used in the design?

9. How did this concept make you feel about contraception? (nondaily and long-acting reversible contraception?)

10. In terms of emotions, how did this concept or message make you feel? In other words, how angry, frustrated, happy, pleased, or sad did you feel?

11. What, if anything, would you change about this message to increase use of nondaily or long- acting reversible contraception?

12. How, if at all, did viewing this message impact your opinion of these contraceptive methods?

*Probe:* Based on this message, would you be more likely to consider using a nondaily or LARC method?

*Probe:* Based on this message, do you plan to contact your health care provider to inquire about these options?

[Repeat 4-12 for each message concept and design]

13. How, if at all, would the sponsoring organization impact your assessment of the message concepts and designs you just saw?

*Probe:* Can you think of a sponsoring organization that would increase/decrease credibility of the message?

---------------------------------

14. So those are all the messages I have to share with you. Now that you have seen all four, how would you compare or contrast them in light of what they are trying to do?

*Probe:* How do they connect with you?

*Probe:* What is on your mind about contraception? (Would you consider switching to a nondaily or LARC method? If so, when?)

15. Those are all the questions I have. Can you think of other questions I should have asked, about the messages or your opinions on them, that I did not?

16. Are there any final comments you would like to share?
